# Supplementary figures and images for: Automated image analysis to assess hygienic behaviour of honeybees
Source: PLoS One. 2022 Jan 27;17(1):e0263183. doi: 10.1371/journal.pone.0263183 (PMC8794212; doi:10.1371/journal.pone.0263183)

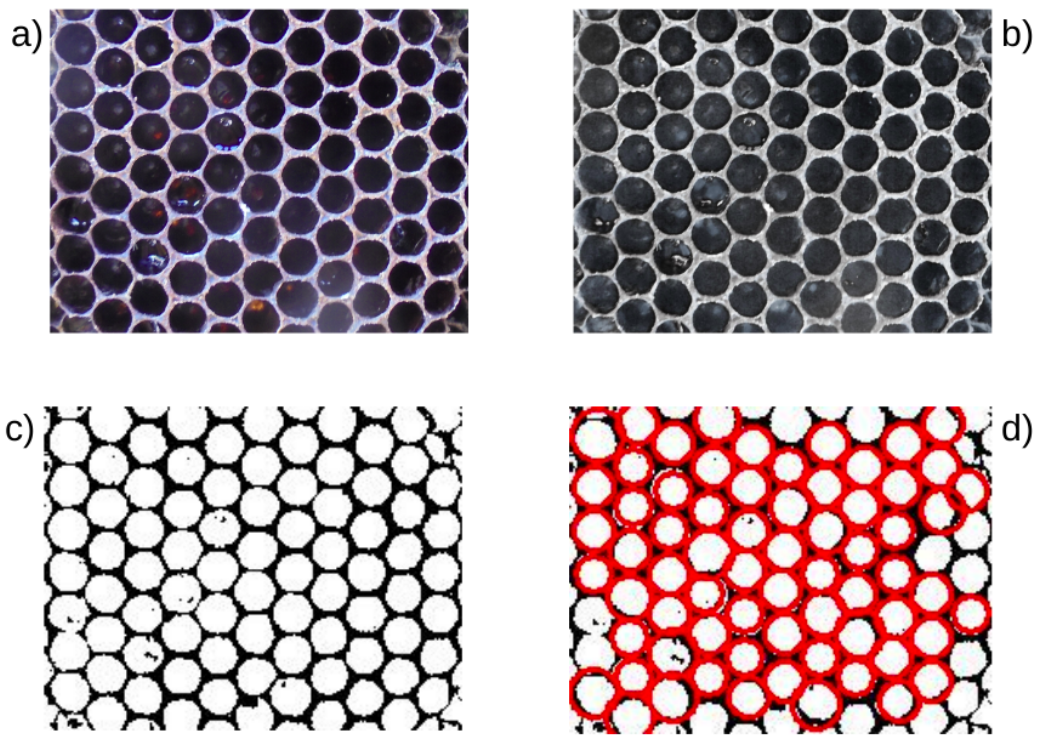

Supplement: S1 Fig — Original crop image a), normalized image b), thresholded image c), circle-detected image d). (TIF) [file pone.0263183.s001.tif]
