# Supplementary material for: Automated image analysis to assess hygienic behaviour of honeybees
Source: PLoS One. 2022 Jan 27;17(1):e0263183. doi: 10.1371/journal.pone.0263183 (PMC8794212; doi:10.1371/journal.pone.0263183)
Supplement: Attachment — Submitted filename: Answers_to_Rev.pdf [file pone.0263183.s004.pdf]

Authors' answer to Editor's and Reviewer's comments

Dear Editor and Reviewer,

We thank you for the interest in our work and for your notes that surely helped us to improve the scientific quality of our manuscript.

Please find below detailed answers to your comments.

Yours sincerely

elena casiraghi (on behalf of all authors)

Reviewer's Responses to Questions

1. Is the manuscript technically sound, and do the data support the conclusions?

Reviewer #1: Yes

---

2. Has the statistical analysis been performed appropriately and rigorously?

Reviewer #1: Yes

---

3. Have the authors made all data underlying the findings in their manuscript fully available?

Reviewer #1: Yes

---

4. Is the manuscript presented in an intelligible fashion and written in standard English?

Reviewer #1: Yes

Reviewer #1: *In the manuscript "Automated image analysis of Varroa related traits in honeybee comb images" a new approach for the automated counting of open/capped brood cells of honeybees is presented and applied to images from a freeze killed brood (FKB). The manuscript presents a new method and is definitively worth publishing, however, the title and abstract seem to fit very well to the content of the manuscript, as title and abstract suggest that the paper focuses on analyses of FKB test in relation to Varroa infestation, while in fact the paper is a very technical manuscript focussing almost exclusively on image recognition algorithms. Therefore, I'd suggest changing the title and rewriting the abstract to make it clearer what the paper is about. I would also mention the highlight of the new method in the abstract, i.e. the use of methods which can cope with very varying image quality or exposure.*

Authors: We would like to thank the Reviewer for his notes. We have carefully revised our manuscript to address all his/her concerns. Briefly, as the Reviewer suggested we have revised both the title and abstract to make clear that we are presenting an automated cell counting method whose strength is the ability of coping with varying image quality or exposure.

Please, find below the detailed answers to all the Reviewers' concerns.

Specific comments:

Line 29: *The sentence "Focus of this study is to design an automated pipeline for segmentation and automatic count of honeybee comb images acquired during summer 2020 by beekeepers." is not clear to me. What do you mean with segmentation? And I guess you don't want to count images, but maybe cells?*

Authors: we agree with the Reviewer the the sentence was not clear. In the digital image processing field, "segmentation" refers to the automatic identification of objects in the image. We changed the sentence to better clarify that our aim is the identification of capped brood cells in honeybee comb images, which ultimately allows their automatic counting.

Line 34: *What is meant with "To recover from acquisition"? Do you mean for image optimisation? Or for normalisation?*

Authors: we tested three different image enhancement algorithms to choose the one that mostly reduced the effect of bad illumination conditions or exposures. We agree with the Reviewer that our sentence was not clear and we therefore substituted that sentence with the following one:

"[...] To reduce the effects of varied illuminations or exposures, three image enhancement algorithms were tested and compared […]"

Line 37 and Line 47: *From the beginning of the abstract I expected that varroa infestation is addressed. From the sentence here it seems that only cells are counted. That is confusing. What is the subject of the paper?*

It seems that indeed only cells are counted. Then I find the title a bit misleading. The title could be e.g. "Automated image analysis to assess hygienic behaviour of honeybees".

Authors: The subject of the paper is the automatic count of capped brood cells in honeybee comb images acquired under varied illuminations and exposures and we agree with the Reviewer that the title we used was misleading while the one She/He suggests is more appropriate. We therefore changed the title as suggested by the Reviewer.

Lines 39 and 43: *What is the difference from the 77 "unseen" images and the 100 randomly extracted images? Both were taken from the set of 127 images after removal of the images used for training (but then the numbers wouldn't add up)? Or did you mix images used for training and images not used for training in the set of 100 images? If so, that would not be suitable for validation. I suggest to clarify this section.*

Authors: we are sorry but indeed the abstract was confusing. Our dataset contains 127 images acquired under various illuminations and exposures. To obtain an unbiased evaluation, we randomly split our dataset to obtain a training image set (composed of 50 images) and a test image set (composed of 77 images).

The training images have been used for developing the pipeline and for parameter tuning, that is for choosing the parameters values that allow to obtain the best performance on the training set.

The test image set allows obtaining an unbiased estimate because the images in the test set were never used during the pipeline development or the parameter tuning phase. This is why they are generally called "unseen" in the artificial intelligence field. Anyhow, to avoid confusion, we have removed the adjective "unseen" in the revised abstract.

On the other hand, the dataset composed of 100 images is a completely different dataset that has been randomly extracted from the publicly available dataset by Alves et al. We have used it for assessing our model against a publicly available dataset.

We have rewritten the Abstract to remove all the confusing sentences. More precisely, the following sentences summarize how we split our dataset into training and test sets and that we also tested our model on an external, dataset randomly extracted from a publicly available dataset:

[...] To obtain an unbiased evaluation, the cropped images were randomly split into a training image set (50 images), which was used to develop and tune the proposed model, and a test image set (77 images), which was solely used to test the model. [...]

When applied to the 77 test images the model obtained a correlation of 0.819 between the automated counts and the experts' counts. We further assessed the proposed model on 100 images randomly extracted from a public dataset acquired under controlled conditions. On this new set, the correlation with manually counted cells on the dataset was much higher (0.997) than the one we obtained on our dataset. [...]

Line 68: *The list of references given could be more comprehensive. There are more papers on counting brood and uncapped cells and papers where such methods have been applied: Jeker et al. 2012, Avni et al. 2015, Cutler et al. 2014, Colin et al 2018, Wang er al. 2020, etc.*

Authors: we thank the reviewer and we have added all the above mentioned references in the introduction of the revised manuscript.

Line 81: *Controlled conditions are generally used in order to optimise results in particular when doing large scale analyses. It is not so much the problem that automated analysis doesn't work well if conditions are not controlled, but that manual adjustment of images is not needed when using controlled conditions. Hence it is more a practical point to reduce workload.*

Line 82: *I disagree that it is difficult to do in practice. Most researchers use a setup with artificial light for photography. With that it is easy to produce many images with identical exposure and size of frames in images. Routine analyses can include hundreds to thousands of standardised images. This has also been done in some of the references given above.*

Authors: we agree with the Reviewer.However, the poor results we obtained on our images when running preliminary experiments with CombCount method (Colin at al., 20218 [17]) and the method from Alves et al., 2020 [18] showed that the usage of predefined acquisition settings results in ad-hoc techniques specifically developed for handling the illumination and exposure conditions defined by the acquisition settings.

We have substituted the sentence at lines 81-82 to clear this point.

Line 81: *Furthermore, for counting cells all software solutions seem to work pretty well even under varying conditions. Detecting larvae or eggs is the main problem in practice.*

Authors: We agree with the Reviewer and indeed this is one of our future works. To clear this point we have inserted a sentence at the end of the discussion section.

Line 100: Just a comment, the paper addresses a method to make counting less time consuming. In the study presented in the manuscript images were manually cropped. Would existing software have been used, then both cropping and automated counting could have been done within the software much less time effort.

Authors: as mentioned above, before developing the model we are presenting we performed preliminary experiments with CombCount [17] and the method from Alves et al. [18]. Unfortunately, both the methods provided poor cropping and automated counting results. This is probably due to the fact that our images present different illuminations and exposures.

Lines 95-389: *A general comment on "Materials and methods" and "Results". These sections are very, very detailed and extremely difficult to follow. I expect that only a handfull of readers will be able to understand all detail. The broad majority of those working on honeybees probably want to understand the key principles, but that is difficult to understand for a non-expert in image recognition. The best summary of the paper is figure 1. I would suggest to make these key principles clearer in the text and also to indicate clearly when technical details start (so a non-expert can skip them) and when they end. This could be done by an introductory paragraph in each section, which explains only the key principle in common language and going into detail only after that.*

Authors: we must really thank the Reviewer for the note because it allowed us to greatly improve readability.

To this aim, at the beginning of the "Methods" subsection and of the "Result" section we have inserted introductory paragraphs using common language to summarize the key principles and the technical details that can be found in the section. In this way, non-expert readers may decide whether to skip the Section or to read through it.

Line 115: *Please indicate version numbers of software or libraries.*

Authors: we used version 3.7 of Python and the OpenCv3 v.4.0 package. We indicated those versions in the manuscript.

Line 396: *Maybe I misunderstood the sentence, but I don't think Alves et al (2020) is the first study using completely automatic digital photography. It is being used since over 10 years. Publications are available.*

Authors: the sentence may have been misleading. We rephrased it to make it clear that "[...] The study proposed in Alves et al. is the most recent and promising work proposing a fully automated approach for the detection of capped brood in comb frames in the hive and the classification of seven different comb cell classes [14] [...]"

Line 405: *After reading the methods and result, my impression is that the method has been used to exemplarily assess hygienic behaviour. However, the key finding is the development of a new method based on Hugh transforms and normalisation of images in order to be able to use images with very varying quality or exposure. This is certainly worth publishing, but the title and abstract makes the reader expect a very different paper. Hence I suggest to make clear (in both the title and abstract) that this paper is on a new methodology and that the focus is to use non-standardised images. Then in the methods and results it could be mentioned that it is tested exemplarily on images of a FKB test.*

Another question is, why have many rather standardised images been used for validation if this method is proposed to handle images with very variable exposure? This should also be discussed.

Authors:

We thank the Reviewer for its important note. To clear this point we changed the title as suggested in a previous note, we completely revised the Abstract and we also modified the end of the Discussion section to highlight the strengths of our method, by pointing out that the count of uncapped cells from comb images is an application that shows the ability of our strategy to handle uncontrolled image conditions.

Regarding the experiment on the 100 images randomly extracted from the dataset made publicly available from Alves et al. [14], we provide this further test for two reasons:

1) we wanted to assess the generalizability of our model with respect to the usage of different acquisition equipment and conditions; to this aim, we chose the dataset from Alves et al. [14] because it is the most recent, publicly available dataset, and contains images with varied illuminations even though the image acquisition and exposure are controlled;

2) we believe it is fair to provide results on a set of images that can be also downloaded by the readers.

We cleared this point in the Abstract, in the Methods section and in the Discussion Section.

Again, we would like to thank the Reviewer and Editor for the interest in our work.
