# Supplementary material for: Automated image analysis to assess hygienic behaviour of honeybees
Source: PLoS One. 2022 Jan 27;17(1):e0263183. doi: 10.1371/journal.pone.0263183 (PMC8794212; doi:10.1371/journal.pone.0263183)
Supplement: Attachment — Submitted filename: Answers_to_Rev.pdf [file pone.0263183.s005.pdf]

Authors' answer to Editor's and Reviewer's comments

Dear Editor and Reviewer,

We thank you for the interest in our work and for your notes that surely helped us to improve the scientific quality of our manuscript.

Please find below detailed answers to your comments.

Yours sincerely

elena casiraghi (on behalf of all authors)

Review Comments to the Author

Reviewer #1: *I'd like to thank the authors for the work they put into the revision. The readability and clarity has significantly been improved, which now makes the manuscript more accessible to a wider audience.*

There is only one minor comment:
On Page 4 you compare several existing methods for the automatic counting of brood cells. However, in your response to the reviewers comments you mentioned that you only tested CombCount (Colin at al., 20218 [17]) and the method from Alves et al. (2020 [18]). Hence it is not clear how you concluded on the difficulties of the other methods, if you haven't used these. I would suggest to rephrase the sentence making that clear, e.g. by writing the pipeline by Jeker "seems to" be laborious or IndiCounter "seems to" to depend on specific acquisition conditions (our observation is, by the way, different: We find that in general automated recognition works better when image is ok and in particular capped brood cells are recognised well with all kinds of software, without "specific acquisition conditions"; capped cells are well recognised even in very low-res images from low-end cameras of phones). You could rephrase the sentence to:

"Many software packages, able to evaluate comb frames are available. Some of them [11,12] perform statistical analysis to study the condition of honeybee colony by using commercial softwares ("IndiCounter, WSC Regexperts" available at https://wsc-regexperts.com/en/software-and-databases/software/honeybee-brood-colonyassessment-software/) which seems to be designed for larger scale studies where specific acquisition conditions or often used. On the other hand, the semi-automated pipeline introduced by Jeker et al. [13] seems to requires a laborious acquisition setting that depends on several camera parameters to be carefully set."

Author's answer: we agree with the Reviewer's note and we thank him for suggesting a new sentence, which we used to substitute our old sentence.

Thanks again for the interest in our work.

Yours sincerely

elena casiraghi on behalf of all the authors
